# Coal-tar and Some of Its Medicinal Products1A paper read before the Veterinary Medical Society, Ontario Veterinary College, Toronto, Canada.

**Published:** 1895-03

**Authors:** W. L. West

**Affiliations:** Student


### COAL-TAR AND SOME OF ITS MEDICINAL PRODUCTS.[1]

BY W. L. WEST, SR., STUDENT.

Mr. President and Fellow Students: To write a complete description of coal-tar and its products would require the outlay of considerable time, and a large volume would result. My

[1] A paper read before the Veterinary Medical Society, Ontario Veterinary College, Toronto, Canada.

purpose this evening is but to bring to your notice a few of its compounds, which I have thought most useful to us as veterinarians.

When coal-tar is subjected to destructive or dry distillation besides illuminating gas and coke there are found numerous other substances, varying in amount and character according to the quality of the coal and the manner of its decomposition. These substances are volatile, boiling at varying temperatures, and originally condensed into a thick, black liquid, the familiar coal tar. Formerly this was a waste product and a source of considerable loss and trouble to the gas manufacturer. It is now, through the discoveries of chemists, a most valuable bye product, and the source of an infinite series of compounds of interest in the arts, science, and medicine.

Those especially applicable in veterinary practice are naphthalene, methyl-naphthalene, diphynel, retene, which are solids. Benzine, toluene, mesitylene are liquids, while among the acid compounds we have phenol, orthocresol, paracresol, metacresol, phlorol, rosolic acid, and creosote.

PHENOL—PHENYLIC ALCOHOL, CARBOLIC ACID, is one of the most useful and widely-known products of coal-tar. It was discovered and isolated in 1834 by Rungé, who gave it its name, but the honor of introducing it to the profession belongs to Calvert.

NAPHTHALENE occurs as a white, shining, crystalline solid, melting at 176° F.; boils at 423° F. It is soluble in ether, alcohol, chloroform, and the oils. Insoluble inwater, but imparts its taste and odor to water in which it has been boiled for some time. Naphthalene is toxic to all fungi and micro-organisms, and for this reason is a valuable antiseptic. Manufactured now on a very large scale, it is consequently cheap, a point of considerable importance in our practice. It is of value in skin diseases, especially those of a parasitic nature, such as mange, scabies, favus, etc. As an expectorant in bronchitis and other respiratory diseases it has proven very useful. Dose for a horse, ʒss to ʒj.

NAPHTHOL—$\beta$-NAPHTHOL occurs in coal-tar, but is usually prepared by direct methods from naphthalene. It is a yellowish-white powder, possessing the well-known phenol odor and pungent taste. It is permanent in the air, and soluble in 1000 parts of cold and 75 parts of boiling water, and soluble in all parts of alcohol, ether and oils.

Its antiseptic and physiological actions were discovered and investigated by Bouchard, who found that a strength of $\frac{1}{3000}$ solution was sufficient to arrest the growth of pathogenic cultures in an agar tube, and as it is practically non-poisonous (the toxic dose for a man being 10 ounces) is, I think, a valuable remedy which would be useful in diarrhœa, dysentery and influenza where the alimentary tract is involved. Kaposi recommends a solution in oil of 1 to 250 as highly useful in conjunctivitis, laryngitis, etc. In man it has been used with much success in la grippe, and in cases of influenza in our patients might also prove of value, being administered by intertracheal injections of a 1 to 1000 solution in water. Dose for horse, grs. xv. to ʒj.

Salol—Salicylic Ether of Phenol. This was first introduced to the profession by Dr. Sahli, of Basel. It occurs in white shining powder, tasteless, with faint aromatic odor. It is soluble in alcohol, ether and oils, but insoluble in water. It is analgesic and antipyretic, but as an anti-rheumatic it is neither so safe nor so effectual as salicylic acid. On account of its non-irritant properties it forms a useful external dressing. Dose for horse, ℥iv to ℥vij.

Creolin. An emulsion of cresol obtained by means of resin soap. This was first experimented with by Esmarch, who states that as a germicide it is more valuable than carbolic acid, excepting in the case of the bacillus anthracis; creolin is said to be non-poisonous, but this must not be taken in its widest sense, as cases of poisoning have occurred by its careless and indiscriminate use, but compared to most antiseptics it is well nigh innocuous. Its actions and uses internally are only just beginning to be appreciated. It prevents too rapid fermentation, and is useful in tympanites, flatulent colic, acute indigestion, etc., and as a vermicide, either in drench or as an enema, it is very certain and speedy in its results.

It is one of the most useful of agents for rendering instruments aseptic without corroding the metal, as so many antiseptics do. Mixed with iodoform it destroys the odor without affecting its value. Dose for horse, ʒij to ʒvj.

Lysol. This is prepared by dissolving the fraction of coal-tar which boils between 190° C. to 200° C. in fat, and subsequently emulsifying with alcohol. It is a clear, oily liquid, with a feeble aromatic odor, and contains 50 per cent. cresol; is miscible with water, alcohol, ether, and oils. Lysol was first introduced by Dr. Gerlach in June, 1891. After elaborate research he found that both in mixed masses and pure cultures of pathogenic organism it proved a better germicide than carbolic acid or creolin. A 1 per cent. solution has a soapy feeling, and is useful for rendering the hands aseptic without injury. It does not affect the steel instruments, but will ruin celluloid handles. Wounds may be sprayed by a 3 per cent. solution, but a weaker one would answer all purposes. It has no poisonous properties.

There are very many more useful medicinal compounds derived from coal tar, but I think those named will suffice for the present.

